# Postprandial oxidative stress in response to dextrose and lipid meals of differing size

**DOI:** 10.1186/1476-511X-9-79

**Published:** 2010-07-27

**Authors:** Richard J Bloomer, Mohammad M Kabir, Kate E Marshall, Robert E Canale, Tyler M Farney

**Affiliations:** 1 Cardiorespiratory/Metabolic Laboratory, Department of Health and Sport Sciences, University of Memphis, Memphis, TN, USA

## Abstract

We have recently noted that ingestion of dietary lipid (in the form of heavy whipping cream) leads to greater oxidative stress than dietary carbohydrate (in the form of dextrose), when consumed in isocaloric amounts.

**Objective:**

In the present investigation we attempted to replicate our work and also to determine the oxidative stress response to dextrose and lipid meals of two different kilocalorie (kcal) amounts.

**Design:**

Nine young (22 ± 2 years), healthy men consumed in a random order, cross-over design one of four meals/drinks: dextrose at 75 g (300 kcals), dextrose at 150 g (600 kcals), lipid at 33 g (300 kcals), lipid at 66 g (600 kcals). Blood samples were collected Pre meal, and at 30 min, 60 min, 120 min, and 180 min post meal. Samples were assayed for glucose, triglycerides (TAG), malondialdehyde (MDA), and hydrogen peroxide (H_2_O_2_). Area under the curve (AUC) was calculated for each variable, and a 4 × 5 ANOVA was utilized to further analyze data.

**Results:**

A meal × time effect (p = 0.0002) and a time effect was noted for glucose (p < 0.0001; 30 min > Pre, 1 hr, 2 hr, and 3 hr). The dextrose meals primarily contributed to this time effect. No other effects were noted for glucose (p > 0.05). A meal effect was noted for TAG (p = 0.01; 66 g lipid meal > 75 g and 150 g dextrose meals). No other effects were noted for TAG (p > 0.05). An AUC effect was noted for MDA (p = 0.04; 66 g lipid meal > 75 g and 150 g dextrose meals). A meal × time effect (p = 0.02) and a meal effect was noted for MDA (p = 0.004; 66 g lipid meal > 75 g and 150 g dextrose meals). No time effect was noted for MDA (p = 0.72). An AUC effect was noted for H_2_O_2 _(p = 0.0001; 66 g lipid meal > 33 g lipid meal and 75 g and 150 g dextrose meals). A meal × time effect (p = 0.0002), a meal effect (p < 0.0001; 66 g lipid meal > 33 g lipid meal and 75 g and 150 g dextrose meals), and a time effect was noted for H_2_O_2 _(p < 0.0001; 2 hr > Pre, 30 min, and 1 hr; 3 hr > Pre). The time effect for H_2_O_2 _was primarily influenced by the 66 g lipid meal.

**Conclusions:**

These data indicate that 1) minimal oxidative stress is observed following ingestion of dextrose loads of either 75 g or 150 g, or a lipid load of 33 g and 2) lipid ingestion at 66 g leads to greater oxidative stress than lipid at 33 g or dextrose at either 75 g or 150 g. Hence, in a sample of young and healthy men, only 66 g of lipid (taken in the form of heavy whipping cream) leads to a significant increase in blood oxidative stress, as measured by MDA and H_2_O_2_.

## Introduction

The process of normal cellular metabolism leads to the production of reactive oxygen species (ROS), the majority of which are inactivated by endogenous and exogenous antioxidants [1]. The balance between ROS production and antioxidant defense determines the intracellular redox environment, which is responsible for the initiation/regulation of multiple physiological processes within living systems, controlling functions such as cell signaling, DNA and RNA synthesis, protein synthesis, enzyme activation, and apoptosis [2,3].

In general, a low-grade production of ROS (i.e., more reducing conditions) is associated with enhanced health; whereas, excessive ROS production and a chronic oxidative shift in the redox environment has been implicated in a wide variety of pathological conditions [4]. This later scenario is commonly referred to as oxidative stress [1] and occurs when ROS production is increased and/or antioxidant defenses are decreased. This is well described under conditions of increased environment stress [1] and physical stress [5,6], as well as following ingestion of high carbohydrate and high fat meals in which increased substrate metabolism occurs [7]. These and other ROS-generating stimuli can result in oxidative damage to nucleic acids, lipids, and proteins, which has the potential to contribute to the development of human disease [8]. In fact, oxidative stress is suggested to play a primary or secondary role in the pathophysiological mechanisms of multiple acute and chronic human illnesses/diseases [8].

In relation to the ingestion of food, oxidative stress has been reported to occur during the minutes to hours following intake. This "postprandial oxidative stress" is likely a result of both a dramatic increase in ROS production, coupled with a decrease in antioxidant defense (commonly measured in blood samples of human participants) [7]. Following ingestion of carbohydrate and lipid rich meals, an acute state of hyperglycemia/hypertriglyceridemia may result, which is evidenced by an increase in circulating blood glucose, free fatty acids, and triglycerides [9]. This excessive influx of substrate, either within the circulation and/or peripheral tissues, appears to be associated with an increased leakage of electrons from within the mitochondrial respiratory chain, thereby resulting in accelerated superoxide generation [10-12]. Superoxide (possibly working through the activation of nuclear transcription factor-κβ [13]) appears to stimulate a harmful biochemical cascade throughout the circulation, which induces inflammation, endothelial dysfunction, hypercoagulability, and sympathetic hyperactivity, all of which may promote further ROS generation and oxidative damage [9].

The postprandial production of ROS appears highly correlated to both the glucose [14] and the TAG response to feeding [15-18]. This is evident by reported increases in blood biomarkers of oxidative stress during the postprandial period. Multiple studies have used either independent administration of a lipid [16-25] or carbohydrate rich meal [22,26-34] to induce oxidative stress.

In terms of comparisons between carbohydrate and lipid meals, we have recently noted that ingestion of dietary lipid (in the form of heavy whipping cream) leads to significantly greater oxidative stress than dietary carbohydrate (in the form of dextrose), when these are consumed in isocaloric amounts [18]. Our subsequent work indicates that carbohydrate in the form of either dextrose or maltodextrin does not result in a significant amount oxidative stress, even when consumed at an amount equal to 2.25 g per kg body mass by healthy men [35]. Together, these findings provide evidence to support the notion that high fat diets may be more detrimental to overall health than high carbohydrate diets (at least with regards to young, otherwise healthy men). However, it remains unknown what the effect of meals that differ in kcals have on postprandial oxidative stress. While it is logical to assume that higher kcal meals of the same nutrient will result in greater oxidative stress, to our knowledge, no study to date has determined this. It is possible that a ceiling effect occurs for ROS production and subsequent oxidative stress, in that consumption of nutrients above a certain amount may not lead to further ROS production. To address this question, the present study was designed to 1) replicate our initial work demonstrating a greater oxidative stress for lipid compared to isocaloric carbohydrate feedings, and to 2) determine the effect of carbohydrate and lipid meals of two different caloric contents on postprandial oxidative stress. We hypothesized that the lipid meals would result in greater oxidative stress as compared to the dextrose meals and that the degree of oxidative stress would be dependent on meal size (larger meals = greater oxidative stress).

## Methods

### Subjects

Ten young, healthy men were recruited from the University of Memphis and surrounding community and completed all aspects of this study. Sample size was chosen based on our prior work and the work of others focused on postprandial oxidative stress using similar outcome variables. All subjects were non-smokers, of normal weight, normolipidemic (fasting triglycerides < 200 mg·dL^-1^), non-diabetic (fasting glucose < 126 mg·dL^-1^), not regularly using antioxidant supplements or drugs, and did not have diagnosed cardiovascular or metabolic disorders. It should be noted that only nine subjects successfully completed all meal testing. Subject descriptive characteristics are presented in Table 1.

**Table 1:** Characteristics of 9 men.

Variable	Value
Age (yrs)	22 ± 2
Height (cm)	181 ± 8
Weight (kg)	82 ± 12
BMI (kg·m^-2^)	25 ± 4
Body fat (%)	19 ± 7
Waist (cm)	84 ± 9
Hip (cm)	103 ± 6
Resting heart rate (bpm)	68 ± 10
Resting SBP (mmHg)	117 ± 6
Resting DBP (mmHg)	66 ± 9
Fasting glucose (mg·dL^-1^)	102 ± 14
Fasting triglycerides (mg·dL^-1^)	90 ± 50

Data are mean ± SD.

Health history, drug and dietary supplement usage, and physical activity questionnaires were completed by subjects to determine eligibility. Prior to participation, each subject was informed of the procedures, potential risks, and the benefits associated with the study. This was done through both verbal and written form in accordance with the approved procedures of the University Institutional Review Board for Human Subjects Research. Subjects signed an informed consent form (approval # H10-09) prior to be being admitted as a subject.

### Subject Screening (initial laboratory visit)

During the initial visit to the laboratory, subjects completed the informed consent form, health form, and physical activity questionnaire. The height, weight, and body composition (via 7 site skinfold assessment) of each subject was measured using a stadiometer, digital scale, and Lange skin fold calipers, respectively. Heart rate (via palpation) and blood pressure (via auscultation) were recorded following a 10 minute period of quiet rest. An explanation of dietary data recording was provided, along with data collection forms.

### Meal Testing

All subjects who met study criteria reported to the laboratory in the morning following a 10-hour overnight fast. Subjects rested for 10 minutes and then a pre-meal blood sample was collected. On four different days, in random order cross-over design, and separated by 3-7 days, subjects consumed one of four meals: dextrose at 75 grams (300 calories), dextrose at 150 grams (600 calories), lipid at 33 grams (300 calories), lipid at 66 grams (600 calories). The dextrose was in powder form (NOW Foods, Bloomingdale, IL; 100% carbohydrate kcal; 100% sugar) and the lipid consisted of heavy whipping cream (standard dairy grade; 100% fat kcal; 60% saturated fat, 30% monounsaturated fat, 10% polyunsaturated fat). The 300 kcal drinks contained a total of 350 mL of fluid and the 600 kcal drinks contained a total of 700 mL of fluid. The amount of dextrose powder and whipping cream was weighed (laboratory grade balance) and measured prior to the mixing of each drink. The volume of water added to each drink (in order to bring the total volume to 350 mL or 700 mL) was measured in a graduated cylinder. All portions were mixed in a blender. Subjects were given 10 minutes to consume the assigned drink.

The postprandial observation period lasted three hours, during which time four additional blood samples were collected (30 min, 60 min, 120 min, and 180 min). We have noted in our previous work that in healthy men, the peak oxidative stress response occurs between 2-4 hours following ingestion of a lipid meal [17,18,36]. Therefore, we believed that ceasing the observation period at 3 hours post feeding was justified. Subjects remained in the laboratory during this period (or in close proximity) and expended as little energy as possible. No additional meals or calorie containing beverages were allowed during this period. Water was allowed *ad libitum *during the first test day and matched for all subsequent test days. These procedures are similar to those we have used in several recent investigations [16-18,36-39].

### Blood Sampling and Biochemistry

Venous blood samples (~15 mL) were taken from subjects' forearm via needle and Vacutainer^® ^by a trained phlebotomist. Following collection, blood samples were processed accordingly, and the plasma/serum was immediately stored at -70°C until analyzed. All blood samples were assayed for the following variables: glucose, triglycerides (TAG), malondialdehyde (MDA), and hydrogen peroxide (H_2_O_2_). We have recently studied these same variables in response to isocaloric dextrose and lipid meals (albeit only one size, as opposed to two different sizes in the present design). Therefore, we wanted to have similarity in our measures for the current study.

Assays for serum glucose and TAG were performed following standard enzymatic procedures as described by the reagent manufacturer (Thermo Electron Clinical Chemistry). Standard curves for all assays were developed for determination of unknown samples. MDA was analyzed in plasma using a commercially available colorimetric assay (Northwest Life Science Specialties, Vancouver, WA), using previously described methods [40]. H_2_O_2 _was analyzed in plasma using the Amplex Red reagent method as described by the manufacturer (Molecular Probes, Invitrogen Detection Technologies, Eugene, OR). In the reaction mixture, hydrogen peroxide, in the presence of horseradish peroxidase, reacts with Amplex Red reagent to generate the red-fluorescence oxidation product, resorufin. All assays were performed in duplicate on first thaw. These are commonly performed within our laboratory and the coefficient of variation for all measures is ≤7%.

### Dietary Records

Subjects were instructed to maintain their normal diet, and to record their food and beverage intake during the 24 hour period prior to each test day. Subjects were asked to consume similar food choices and quantities during the 24 hours prior to each test day. Nutritional records were analyzed for total kcals, protein, carbohydrate, fat, vitamin C, vitamin E, and vitamin A (Food Processor SQL, version 9.9, ESHA Research, Salem, OR). Subjects were asked to maintain their normal physical activity habits during the study period but to avoid strenuous exercise during the 24 hours immediately preceding the test days, since such activity may have impacted the chosen biomarkers, as reported previously [41,42].

### Statistical Analysis

For each biomarker, the area under the curve (AUC) was calculated using the trapezoidal method as described in detail by Pruessner et al. [43]. In addition, biochemical variables were analyzed using a 4 (meal) × 5 (time) repeated measures analysis of variance (ANOVA). Significant interactions and main effects were further analyzed using Tukey's post hoc tests. Dietary variables were analyzed using a one-way ANOVA. Pairwise correlations were made between all biomarkers using AUC data. All analyses were performed using JMP statistical software (version 4.0.3, SAS Institute, Cary, NC). Statistical significance was set at P ≤ 0.05. The data are presented as mean ± SEM, except for subject descriptive characteristics which are presented as mean ± SD.

## Results

As indicated earlier, although 10 subjects were enrolled in the study, only nine subjects successfully completed all meal testing. One subject decided not to complete the testing protocol due to loss of interest. Therefore, data are only presented for nine subjects. No statistically significant differences were noted for kilocalories (p = 0.34), protein grams (p = 0.87), carbohydrate grams (p = 0.50), fat grams (p = 0.53), vitamin C (p = 0.76), vitamin E (p = 0.85), or vitamin A (p = 0.73). Data are presented in Table 2.

**Table 2:** Dietary data of 9 men during the 24 hours before intake of a dextrose or lipid meal.

Variable	Dextrose 75 g	Dextrose 150 g	Lipid 33 g	Lipid 66 g
Kilocalories	2023 ± 237	2354 ± 242	1983 ± 206	1789 ± 181
Protein (g)	92 ± 11	102 ± 9	95 ± 13	88 ± 16
Carbohydrate (g)	261 ± 39	315 ± 41	248 ± 31	247 ± 33
Fat (g)	72 ± 11	81 ± 12	72 ± 13	57 ± 9
Vitamin C (mg)	64 ± 26	47 ± 11	40 ± 7	51 ± 13
Vitamin E (mg)	4 ± 2	4 ± 1	3 ± 1	3 ± 1
Vitamin A (RE)	267 ± 82	374 ± 110	228 ± 113	236 ± 102

Data are mean ± SEM.

No statistically significant differences noted for kilocalories (p = 0.34), protein (p = 0.87), carbohydrate (p = 0.50), fat (p = 0.53), vitamin C (p = 0.76), vitamin E (p = 0.85), or vitamin A (p = 0.73).

**Figure 1:**
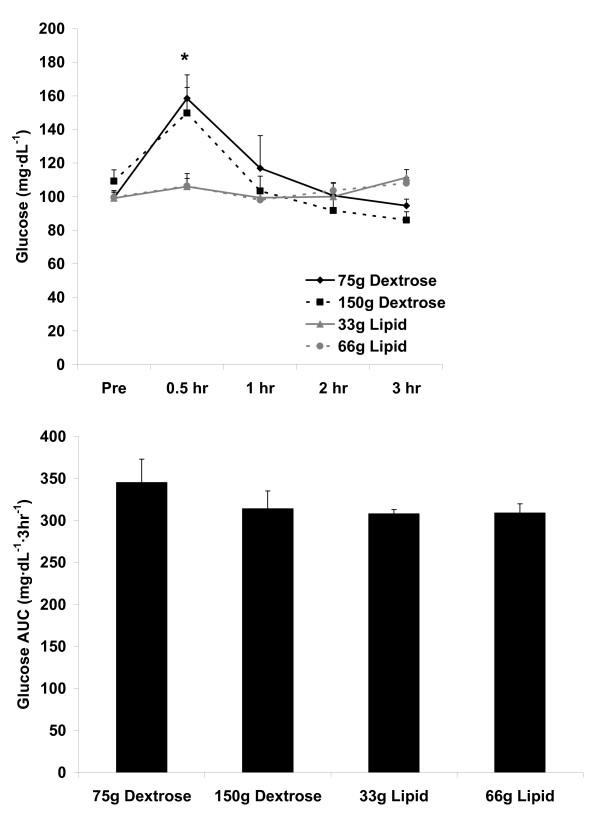
**Blood glucose before and following the consumption of a dextrose or lipid meal in young healthy men**. Data are mean ± SEM. Meal effect (p = 0.13). *Time effect (p < 0.0001); 0.5 hr > Pre, 1 hr, 2 hr, and 3 hr. Meal × Time effect (p = 0.0002). AUC effect (p = 0.44).

For blood glucose, no AUC effect was noted (p = 0.44). No meal effect was noted (p = 0.13), although both a meal × time effect (p = 0.0002) and a time effect was noted (p < 0.0001), with 30 min greater than Pre, 1 hr, 2 hr, and 3 hr (p < 0.05). As expected, the glucose meals contributed most to this time effect. Data are presented in Figure 1.

**Figure 2:**
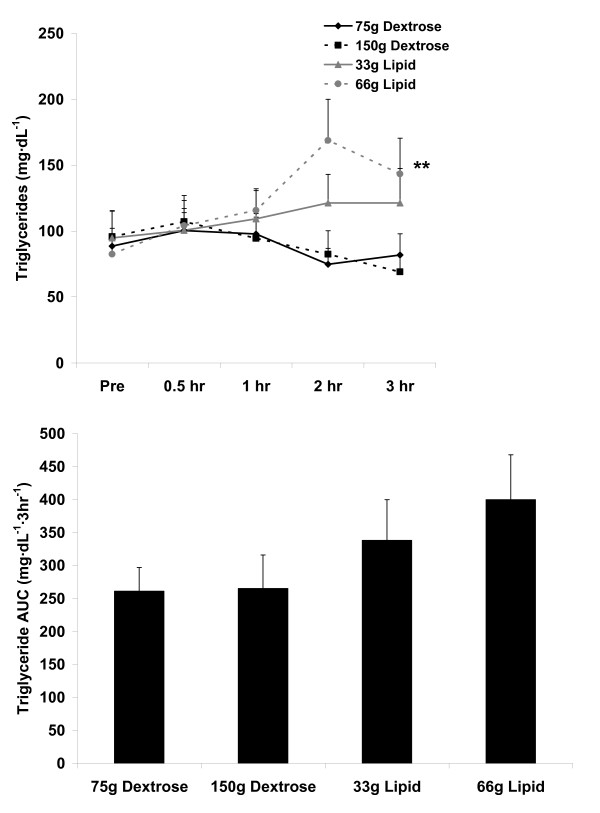
**Blood triglycerides before and following the consumption of a dextrose or lipid meal in young healthy men**. Data are mean ± SEM. **Meal effect (p = 0.01); 66 g Lipid > 75 g Dextrose and 150 g Dextrose. Time effect (p = 0.63). Meal × Time effect (p = 0.27). AUC effect (p = 0.26).

For blood TAG, no AUC effect was noted (p = 0.26). No meal × time effect (p = 0.27) or time effect was noted (p = 0.63). However, a meal effect was noted (p = 0.01), with the 66 g lipid meal greater than the 75 g and 150 g dextrose meals (p < 0.05). Data are presented in Figure 2.

**Figure 3:**
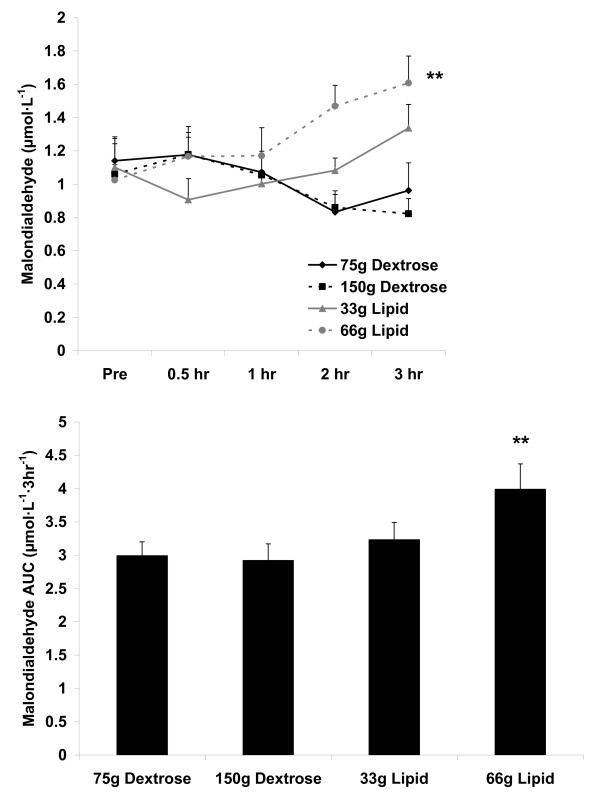
**Blood malondialdehyde before and following the consumption of a dextrose or lipid meal in young healthy men**. Data are mean ± SEM. **Meal effect (p = 0.004); 66 g Lipid > 75 g Dextrose and 150 g Dextrose. Time effect (p = 0.72). Meal × Time effect (p = 0.02). **AUC effect (p = 0.04); 66 g Lipid > 75 g Dextrose and 150 g Dextrose.

For blood MDA, an AUC effect was noted (p = 0.04), with the 66 g lipid meal greater than the 75 g and 150 g dextrose meals (p < 0.05). A meal × time effect (p = 0.02) and a meal effect was noted (p = 0.004), with the 66 g lipid meal greater than the 75 g and 150 g dextrose meals (p < 0.05). However, no time effect was noted (p = 0.72). Data are presented in Figure 3.

**Figure 4:**
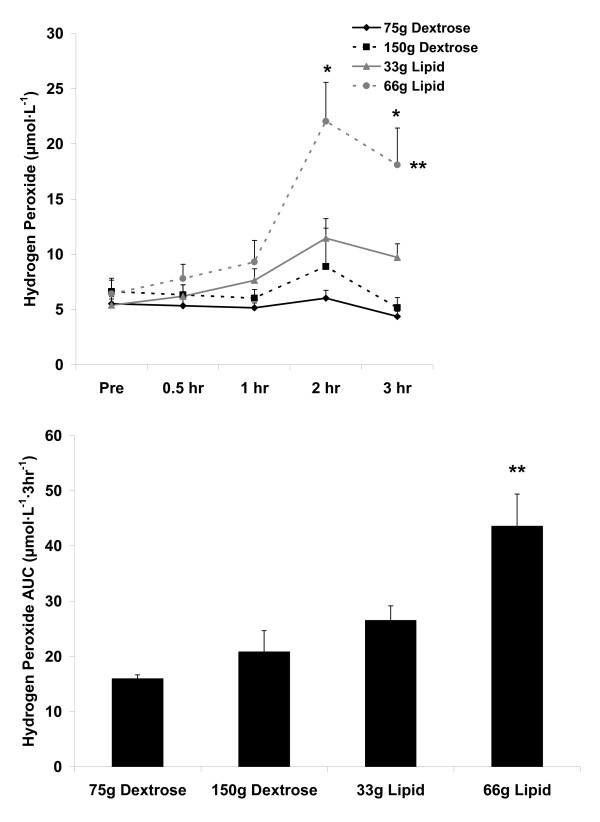
**Blood hydrogen peroxide before and following the consumption of a dextrose or lipid meal in young healthy men**. Data are mean ± SEM. **Meal effect (p < 0.0001); 66 g Lipid > 33 g Lipid, 75 g Dextrose, and 150 g Dextrose. *Time effect (p < 0.0001); 2 hr > Pre, 0.5 hr, and 1 hr; 3 hr > Pre. Meal × Time effect (p = 0.0002). **AUC effect (p = 0.0001); 66 g Lipid > 33 g Lipid, 75 g Dextrose, and 150 g Dextrose.

For blood H_2_O_2_, an AUC effect was noted (p = 0.0001), with the 66 g lipid meal greater than the 33 g lipid meal and the 75 g and 150 g dextrose meals (p < 0.05). A meal × time effect (p = 0.0002), a meal effect (p < 0.0001), and a time effect was noted (p < 0.0001). Regarding the meal effect, the 66 g lipid meal was greater than the 33 g lipid meal and the 75 g and 150 g dextrose meals (p < 0.05). With regards to the time effect, 2 hr was greater than Pre, 30 min, and 1 hr; 3 hr was greater than Pre (p < 0.05). Data are presented in Figure 4.

When considering all four meals combined and using values generated using the AUC analysis, significant positive correlations were noted between many biochemical variables (p < 0.05). A correlation matrix is provided in Table 3.

**Table 3:** Correlation matrix using AUC values for biochemical variables collapsed over all meals.

	Glucose	Triglyceride	Malondialdehyde	Hydrogen Peroxide
Glucose		0.18 p = 0.30	0.03 p = 0.86	0.17 p = 0.35
Triglyceride	0.18 p = 0.30		0.70 p < 0.0001	0.53 P = 0.001
Malondialdehyde	0.03 p = 0.86	0.70 p < 0.0001		0.54 P = 0.0008
Hydrogen Peroxide	0.17 p = 0.35	0.53 P = 0.001	0.54 P = 0.0008	

## Discussion

We noted two main findings from the present investigation. First, minimal oxidative stress is observed following ingestion of dextrose loads of either 75 g or 150 g, or a lipid load of 33 g (Figure 3 and Figure 4). Considering these data, it is clear that even with consumption of a very high load of simple sugar (150 g dextrose), minimal blood oxidative stress occurs (at least as measured using the indirect markers of MDA and H_2_O_2_). This is true despite the transient increase in blood glucose following ingestion of both the 75 g and 150 g dextrose meals (Figure 1). Furthermore, we noted only a small increase in oxidative stress (and TAG) in response to the 33 g lipid meal, indicating that a relatively small lipid load is well tolerated by young, healthy men. Considered collectively, ingestion of simple sugar or moderate amounts of dietary fat (33 g) may not pose any significant health concerns in young, healthy individuals, at least as measured in an acute state. Of course, regular and long-term consumption of such nutrients may result in other potential problems which may not be directly related to postprandial oxidative stress (e.g., weight gain, impaired insulin sensitivity).

Of course, it is possible that an increase in oxidative stress may have been noted following intake of these meals if other biomarkers aside from MDA and H_2_O_2 _were included. It should be noted that besides our previous work, both MDA and H_2_O_2 _have been used by other investigators in previous studies of postprandial oxidative stress: MDA [20,33,44] and H_2_O_2 _[31,45]. A limitation of the present study is the omission of other commonly assessed oxidative stress biomarkers such as protein carbonyls, glutathione (oxidized and reduced), and nitrotyrosine, as well as other specific markers of lipid peroxidation such as F_2_-isoprostanes [8]. Future work may consider the inclusion of these additional assays in an attempt to better characterize the system following ingestion of both dextrose and lipid meals.

In addition to the above, other limitations of this work should be noted. First, it is important to mention that as with most studies in this area of investigation, we only measured *blood *oxidative stress in the present study. Therefore, we cannot assume that oxidative stress did not occur in tissue aside from blood. Second, it should be mentioned that the biomarkers utilized in the present investigation are considered indirect indicators of oxidative stress. Indirect assessment of oxidative stress involves the measurement of the more stable molecular products formed via the reaction of ROS with certain biomolecules [8], as opposed to the direct quantification of ROS. This method is most commonly used throughout the literature, as direct measurement of radical production (via electron spin resonance spectroscopy) is very costly and labor intensive. Despite the regular use of indirect techniques, this could still be considered a limitation of the present investigation. Third, although we noted no differences in dietary intake over the 24 hour period prior to each test day, it is possible that differences in other variables not included in our analysis (e.g., specific antioxidants) could have impacted our findings. Moreover, differences in dietary intake during the *week*, rather than the day, preceding each test day may have influenced our findings. Fourth, our sample size of nine men, although similar to many other studies in the literature, is relatively small and may have impaired our ability to note statistical significance in some instances. Future studies may consider the inclusion of a larger subject sample. Fifth and not necessarily a limitation, our subjects were young and healthy, with most involved in regular physical activity programs. It is possible that older individuals and/or those with known metabolic disorders (e.g., obesity, diabetes) may have responded differently to the meals. Further work should consider the above prior to attempting to generalize results to populations other than young and healthy men, as doing so may be problematic.

Second with regards to our main findings, lipid ingestion at 66 g leads to greater oxidative stress than lipid at 33 g or dextrose at either 75 g or 150 g. These data 1) confirm the results from our prior work in which we noted that ingestion of dietary lipid (heavy whipping cream) leads to greater oxidative stress than ingestion of dietary carbohydrate (dextrose), when consumed in isocaloric amounts [18] and 2) confirm our hypothesis that the magnitude of oxidative stress is dependent on the amount of lipid consumed (66 g > 33 g). However, meal size was not a factor when considering dextrose, as neither the 75 g nor the 150 g dextrose meal resulted in a significant amount of oxidative stress. Again, it is likely that our specific subject sample influenced these findings for both macronutrients, as differing results may have been observed if we included older, overweight, and/or diabetic subjects. Regardless, it is important to note that even in young, healthy subjects who appear to tolerate well, a moderate dose of lipid, as well as moderate to high doses of simple sugar, a high dose of lipid is detrimental in terms of elevating postprandial oxidative stress. Considering the relationship between oxidative stress and the development and progression of human disease [8], routine ingestion of meals rich in saturated fat (consumed as heavy whipping cream in the current study) should be strongly discouraged.

In relation to H_2_O_2 _production, and subsequent formation of MDA, a greater postprandial increase following the lipid meal would be expected based on the commonly accepted mechanisms underlying the regulation of postprandial superoxide production [46]. Considering that superoxide is produced within the mitochondria during substrate oxidation, and because a greater relative supply of electrons downstream to complex I would be expected to be produced as a consequence of accelerated beta-oxidation (via FADH_2 _donation to electron transferring flavoprotein-coenzyme Q oxidoreductase [47]), it follows that the lipid meals (which yield significantly more FADH_2 _than do carbohydrate) would promote greater H_2_O_2 _production compared to the dextrose meals. These findings are in agreement with our work [18] and the work of others [15,32,48], noting greater postprandial oxidative stress following lipid rich meals compared to carbohydrate rich meals. A detailed review pertaining to the role of ROS following ingestion of lipid meals is beyond the scope of this discussion, but can be found in our recent review on this topic [49].

Our results for the dextrose meals parallel some previous findings of minimal increase in oxidative stress when assessed using young, healthy subjects [18,26,29,35,44], but refute other work which has shown an increase in various markers of oxidative stress following a 75 g oral glucose tolerance test [22,26-31]. However, the majority of investigations reporting increased oxidative stress following carbohydrate meals include diabetics as subjects [22,26-30,32-34]. It follows that oxidative stress may be elevated in such individuals, as exacerbations in postprandial oxidative stress have been associated with an insulin resistant state [22,26,29,30]. As stated above, caution should be taken when attempting to extrapolate our present findings to populations other than young, healthy men.

Related to the lipid meals, in particular the 66 g lipid meal, the TAG response to feeding was greater than that of the dextrose meals (Figure 2). Based on the noted correlations between TAG, MDA, and H_2_O_2 _(Table 3), it follows that the lipid meals (in particular the 66 g lipid meal) resulted in a greater oxidative stress as compared to the dextrose meals. However, it should first be noted that the increase in H_2_O_2 _and MDA, although thought to be influenced mainly by the TAG response to feeding, could have been affected by other non-related factors (e.g., *ex vivo *oxidation during the assay procedures). Second, it should be understood that the magnitude and time course of response for TAG, MDA, and H_2_O_2 _does not exactly parallel one another, as can be observed in Figures 2, 3, and 4. It is possible that the early increase in blood TAG (which seems to peak by 2-3 hours post feeding in young, healthy subjects [17,36]) triggers an increase in superoxide which then manifests in an increase in blood H_2_O_2_. Following this rise in H_2_O_2_, lipids undergo oxidation which then leads to the delayed appearance of MDA which can be detected in the circulation. In the present study, MDA was still increasing at the end of the collection period, highlighting the delayed response for this marker of lipid peroxidation, which often occurs between 4-6 hours post feeding. Our failure to extend the collection time beyond three hours may be considered an additional limitation of this work.

The present study is the fourth in which we have shown strong, positive correlations between the TAG response to feeding and the oxidative stress response to feeding [16-18]. Because the measurement of serum TAG is relatively simplistic and cost effective, if oral lipid tolerance tests were considered for inclusion within a clinical setting, the simple measurement of TAG may allow for the prediction of blood oxidative stress biomarkers. Future investigations using a large and diverse subject population may seek to further investigate this relationship and to subsequently develop regression equations which may serve to predict the oxidative stress response to feeding using blood TAG as the primary predictor variable.

In relation to meal size, we did not observe any further increase in either blood glucose or oxidative stress with the 150 g vs. the 75 g dextrose meal. Therefore, we feel confident that simple sugar in the form of dextrose is rapidly and efficiently processed by young, healthy men. However, based on the present design, we cannot rule out that a ceiling effect occurs with lipid in relation to either blood TAG or oxidative stress. There is clearly a greater response with the higher lipid load, and further study is needed with ingestion of even higher amounts of lipid in order to adequately address the question of whether or not a ceiling effect occurs for oxidative stress in response to lipid feedings.

From an applied point of view, we are uncertain that such work will provide practical data. That is, while subjects generally did not have a problem consuming either dextrose meal or the 33 g lipid meal, the 66 g lipid meal was challenging for some subjects--in particular for those who do not routinely ingest high fat meals--and would generally not be consumed in isolation without the inclusion of other macronutrients. However, while this amount of dietary lipid (66 g) can easily be consumed in a mixed meal (whole food or milkshake), it is likely that a similar oxidative stress response may be observed in a non-laboratory based setting where individuals consume high fat, high calorie meals. In a recent study we have compared a pure lipid meal (heavy whipping cream) to an isocaloric mixed meal of lipid, carbohydrate, and protein, noting a much greater oxidative stress response for the pure lipid meal [18]. However, due to the isocaloric nature of the feeding, the total amount of lipid consumed in the mixed meal was less than that of the pure lipid meal. While the isolated lipid and dextrose meals were necessary to address the questions in the present design, we admit that they may not have much application to individuals consuming mixed meals in a free living environment. Therefore, this should be considered in future designs. Although, bearing in mind that the total amount of dietary energy is relatively low even when including the 66 g lipid meal (600 kcal), adding carbohydrate and protein to the lipid load allows for greater practical application, and would likely contribute to a similar or greater oxidative stress response as observed in the present study [16,38,39]. Such a possibility highlights the need to minimize the intake of saturated fat for the purpose of health maintenance.

## Conclusion

The present findings indicate that minimal oxidative stress is observed following ingestion of dextrose at either 75 g or 150 g, or lipid at 33 g. Moreover, lipid ingestion at 66 g leads to greater oxidative stress than lipid at 33 g or dextrose at either 75 g or 150 g. Based on these data we conclude that in a sample of young and healthy men, only 66 g of lipid (consumed in the form of heavy whipping cream) leads to a significant increase in blood oxidative stress. Future research should consider the inclusion of older and metabolically compromised individuals, in an effort to determine their response to such feedings. These studies should consider the incorporation of a more realistic "mixed meal" in terms of macronutrient composition. This should provide for practical applications that may have clinical relevance pertaining to oxidative stress specific ill-health and disease.

## Competing interests

Financial support for this work was provided by the University of Memphis. The authors declare no competing interests.

## Authors' contributions

RJB was responsible for the study design, biochemical work, statistical analyses, and manuscript preparation; REC, MMK, KEM, and TMF were responsible for data collection, blood collection and processing, and assistance with manuscript preparation. All authors read and approved of the final manuscript.
